# Dual-band Tm^3+^-doped tellurite fiber amplifier and laser at 1.9 μm and 2.3 μm

**DOI:** 10.1038/s41598-018-34546-w

**Published:** 2018-11-01

**Authors:** S. V. Muravyev, E. A. Anashkina, A. V. Andrianov, V. V. Dorofeev, S. E. Motorin, M. Y. Koptev, A. V. Kim

**Affiliations:** 1 Institute of Applied Physics of the Russian Academy of Sciences, Nizhny Novgorod, Russia; 2 Center of Laser Technology and Material Science, Moscow, Russia; 3 University of Nizhny Novgorod, Nizhny Novgorod, Russia; 4 G.G. Devyatykh Institute of Chemistry of High-Purity Substances of the Russian Academy of Sciences, Nizhny Novgorod, Russia

## Abstract

Ultrabroadband amplification and two-color CW lasing simultaneously near 1.9 μm and 2.3 μm in a Tm^3+^-doped tellurite fiber were demonstrated experimentally, for the first time to the best of our knowledge. A low-loss Tm^3+^-doped core fiber from TeO_2_–ZnO–La_2_O_3_–Na_2_O glasses stable against crystallization was produced by a special technique, providing a low concentration of hydroxyl groups. Supercontinuum from a highly GeO_2_ doped silica fiber pumped by an Er fiber laser system was used as a seed for an amplifier. A maximum gain of 30 dB and 7 dB was measured at 1.9 μm and 2.3 μm, respectively. We report detailed experimental and theoretical studies, which are in a very good agreement, of laser amplification and generation in the manufactured fiber with carefully measured and calculated parameters. A quantitatively verified numerical model was used to predict power scalability at 2.3 μm in schemes with optimized parameters at increased pump power. The presented results show that a high-quality tellurite fiber is a promising candidate for developing lasers in the 2.3 μm atmospheric window which are particularly relevant for applications in gas sensing, eye-safe laser radars, breath analysis, remote sensing and stand-off trace gas detection.

## Introduction

Laser sources operating in the 2.3 μm atmospheric window are particularly relevant for applications in gas sensing, eye-safe laser radars, breath analysis, biomedicine, remote sensing, and stand-off trace gas detection, especially in oil and gas industry^1^. Laser-based absorption spectroscopy demonstrates high sensitivity trace gas detection with high species selectivity^2^.

A Tm^3+^ ion can provide lasing at a wavelength of about 2.3 μm at the ^3^H_4_ → ^3^H_5_ transition in various matrices^1^. The development of fiber laser systems in the atmospheric window is of great importance because they possess good mass-size characteristics, high quality of the laser beam, good heat dissipation, ease of use, and cost efficiency. However, Tm^3+^-doped silica fibers traditionally used for generation near 2 μm at the ^3^F_4_ → ^3^H_6_ transition in different regimes (see, for example^1,3–6^) do not produce laser action at 2.3 μm at the ^3^H_4_ → ^3^H_5_ transition due to multiphonon relaxation that limits the lifetime of the excited state. It is possible to obtain optical signals at a wavelength of 2.3 μm and longer with Tm^3+^-doped silica fibers via nonlinear pulse conversion^7–12^. However, these schemes usually involve a long chain of pulsed lasers, amplifiers and nonlinear fibers and are more complicated than only a laser at a desirable wavelength. Tm^3+^-doped fluoride fiber lasers at 2.3 μm were demonstrated (see, for example^1–3,13,14^). But fluoride fibers, along with good optical properties, have significant drawbacks such as the tendency to corrosion due to air moisture. Due to low viscosity of fluoride glasses, it is very difficult to completely avoid the occurrence of any crystallization while processing melt through the glass transition (or when drawing a fiber from the melt)^15^. An alternative to Tm^3+^-doped fluoride fibers for laser generation at 2.3 μm could be tellurium dioxide based Tm^3+^-doped fibers. Tellurite glasses have a higher phonon energy, but they are stronger mechanically and possess better chemical stability^16^. Many compositions among the most actively investigated zinc-tellurite and tungstate-tellurite systems are resistant to crystallization^17–19^. Laser generation at 2.3 μm in a bulk Tm-doped tellurite glass sample was recently demonstrated by Denker *et al*^20,21^. It was the first experimental confirmation that a tellurite glass matrix is suitable for lasing at the ^3^H_4_ → ^3^H_5_ transition whereas laser generation near 2 μm at the ^3^F_4_ → ^3^H_6_ transition was previously obtained for different tellurite gain elements such as bulk samples^22^, microspheres^23,24^, and fibers: solid step-index^22,25–27^ and microstructured ones^28^.

**Figure 1:**
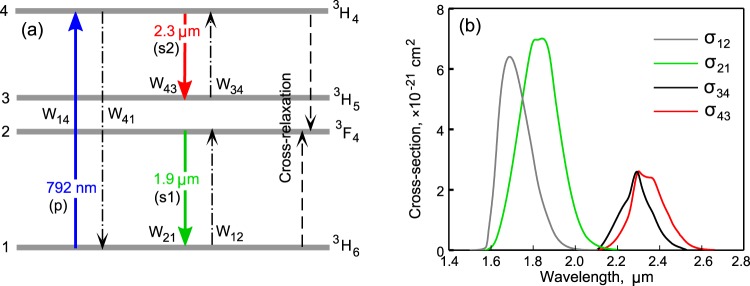
(**a**) Simplified scheme of energy levels of Tm^3+^. (**b**) Emission and absorption cross-sections of ^3^F_4_ → ^3^H_6_ and ^3^H_4_ → ^3^H_5_ transitions.

Obtaining lasing at the ^3^H_4_ → ^3^H_5_ transition is a more complicated task than attaining lasing at the ^3^F_4_ → ^3^H_6_ transition, since the lifetime of the ^3^H_4_ level is an order of magnitude shorter than the lifetime of the ^3^F_4_ level, and the maximum emission cross-section of the ^3^H_4_ → ^3^H_5_ transition is smaller than the maximum emission cross-section of the ^3^F_4_ → ^3^H_6_ transition (see Fig. 1(a,b))^20^. Therefore, when developing novel tellurite glasses and fibers for generation at 2.3 μm, more stringent conditions should be imposed on the quality of the samples in comparison with the samples for generation of only about 2 μm. It is desirable to produce a high-quality fiber with minimum possible background loss, as well as with a low concentration of hydroxyl groups (which can effectively reduce the lifetime of the excited state^29^). In addition, it is necessary to carefully choose the concentration of Tm^3+^ ions. On the one hand, the higher the concentration, the shorter the pump absorption length is and, consequently, the less loss impact. On the other hand, at a high concentration, the cross-relaxation process (^3^H_4_ + ^3^H_6_ → 2·^3^F_4_) becomes significant and leads to depopulation of the upper laser level ^3^H_4_. Therefore, for generating about 2.3 μm special attention should be given to the fiber design.

Here we report experimental demonstration of dual-band ultrabroadband amplification and two-color CW lasing simultaneously near 1.9 μm and 2.3 μm, for the first time to the best of our knowledge, in Tm^3+^-doped tellurite fibers. To do so we developed an ultradry TeO_2_-ZnO-La_2_O_3_-Na_2_O (TZLN) glass stable against crystallization, and the fiber with Tm^3+^-doped 8-μm core on its basis was produced by a special technique, providing a low concentration of hydroxyl groups (~10^17^ cm^−3^) and a low background loss (~1 dB/m in the 2–2.5 μm range). We also developed a numerical model and obtained very good agreement between the experimental and theoretical results. Based on this quantitatively verified model, we optimized numerically laser and amplifier parameters and predicted significant power scaling at 2.3 μm.

## Results

### Development and characterization of Tm^3+^-doped TZLN glass and fiber

We produced an active fiber based on high-purity TZLN undoped glass for cladding and Tm^3+^-doped (with concentration *N*_*Tm*_ = 5·10^19^ cm^−3^) glass for core. The glass system TZLN is characterized by high resistance against crystallization, transparency in the near and mid-IR regions, and good solubility of rare earth oxides. For producing step-index fibers from this glassy system, refractive index can be easily modified by changing the TeO_2_/ZnO concentrations ratio without any significant changes in viscosity, thermal expansion coefficient, crystallization stability, and transmittance. High-purity starting materials: oxide of tellurium TeO_2_ produced by the original technique, oxide of zinc ZnO produced by diethyl zinc oxidation reaction, commercially available lanthanum oxide La_2_O_3_, thulium oxide Tm_2_O_3_, and sodium carbonate were used for preparation of the glasses. The used initial high-purity substances and the technique for preparation of glasses in the closed chamber make it possible to produce tellurite glasses with the total content of 3d-transition metals of less than 0.1–2 ppm wt and with undesirable rare-earth elements concentration less than the detection limit of the laser mass spectrometry (<1–2 ppm wt)^30,31^. The technique of the preparation of high-quality, stable against crystallization, ultradry tellurite glasses was developed previously and described in detail in^17,19^.

**Figure 2:**
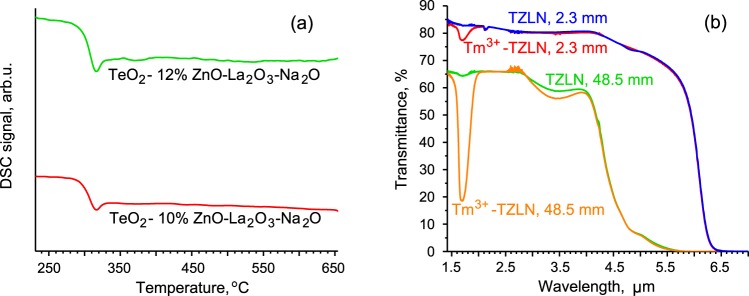
(**a**) Thermograms of DSC of produced TZLN glasses (heating rate 10 °C/min). (**b**) IR transmission spectra of Tm^3+^-doped and undoped TZLN glass samples 2.3 mm long; IR transmission spectra of the whole preform in the core and cladding parts 48.5 mm long.

The NETZSCH STA-409 PC Luxx instrument was used for investigations by differential scanning calorimetry (DSC). Measurements were made in an argon flow with a flow rate of 60 ml/min, at a heating rate of 10 К/min within the temperature range of 200–700 °C. The samples were in the form of pieces polished at the bottom with mass of about 30 mg. The accuracy of the measurement was estimated to be ± 3 °C. Thermal effects in glass were studied by the differential scanning with a heating rate of 10 °C/min. Thermograms of the prepared matrix undoped glasses are presented in Fig. 2(a). The glass transition temperatures of the glasses containing 10 and 12 mol% of zinc oxide are approximately equal to ~300 °C. It can be noted that there are no obvious thermal effects of crystallization and melting of crystals on the given curves at the heating rate applied. The addition of small content of thulium oxide had almost no impact on the glass transition temperature and on the resistance to crystallization for TZLN glasses.

IR spectra were recorded by the IR Nicolet 6700 Fourier spectrometer. The transmission spectra of doped and undoped glass samples of different thicknesses are shown in Fig. 2(b). The minima of transparency near 1.7 μm for Tm^3+^-doped samples are due to ground state absorption at the ^3^H_6_ → ^3^F_4_ transition. There are no apparent absorption bands of hydroxyl groups with typical maxima at 2.3, 3.3, and 4.4 µm on the spectra of short glasses. This indicates a reduced content of hydroxyl groups in the glasses. We calculated hydroxyl groups content to be ~10^17^ cm^−3^ using the same procedure described in detail in^17^.

**Figure 3:**
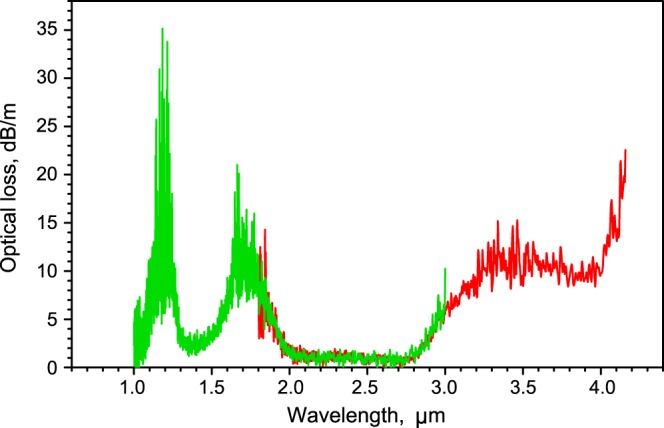
Total optical loss of multimode fiber.

The monolithic preform for fiber production was fabricated by the technique of melt extrusion, the method is similar to that described in^17^. Further, we produced step-index multimode and single-mode (with estimated cutoff wavelength of ~1.95 μm) fibers with core/cladding diameters of 50/157 μm and 8/100 μm, respectively. Optical loss *α* measured in a multimode fiber by the cut-back method using immersion with an indium-gallium alloy is shown in Fig. 3. One can see that the optical loss is of order 1 dB/m in the 2–2.8 μm range. The maximal losses near 1.7 μm and 1.2 μm are due to ground state absorption at the ^3^H_6_ → ^3^F_4_ and ^3^H_6_ → ^3^H_5_ transitions, respectively. The increase of loss at wavelengths beyond 2.8 μm originates from hydroxyl groups absorption. In the experiments on laser amplification and generation, we used only the single-mode fiber.

### Experimental and theoretical study of ultrabroadband amplifier based on Tm^3+^-doped TZLN fiber

**Figure 4:**
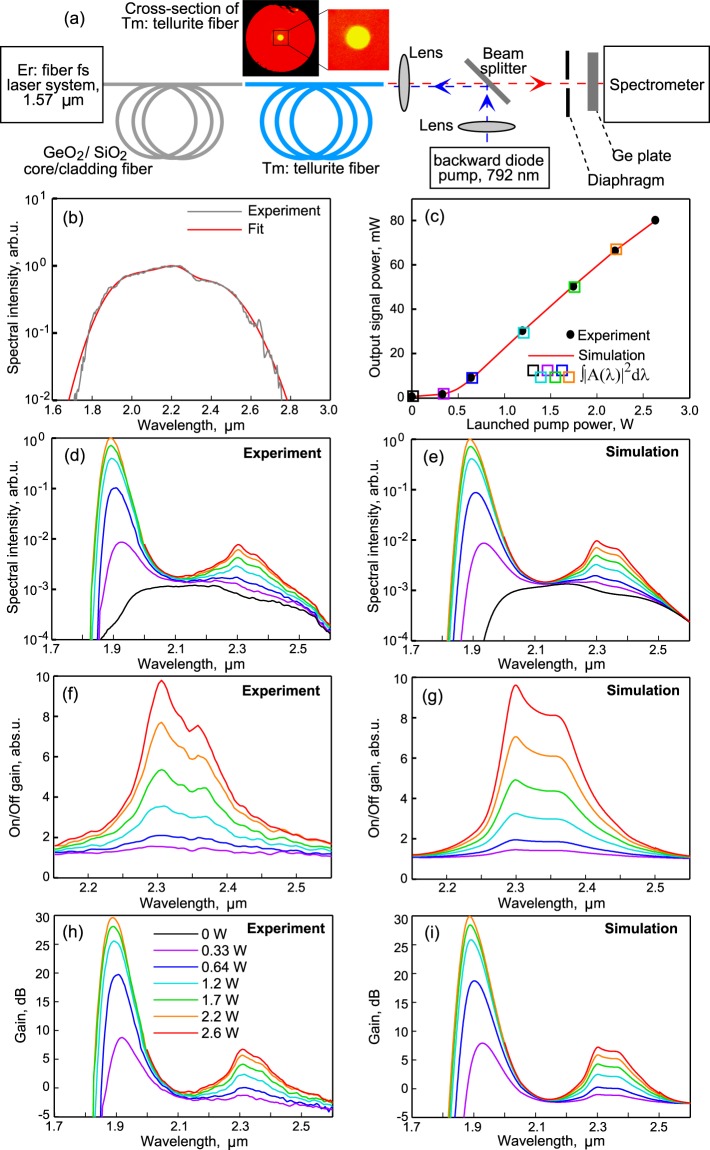
(**a**) Scheme of experimental setup for ultrabroadband amplification together with the image of tellurite fiber cross-section taken from microscope. (**b**) Spectrum of seeded SC. (**c**) Output signal power as a function of pump power. Measured (**d**) and simulated (**e**) spectra of amplified SC. Measured (**f**) and simulated (**g**) On/Off gain (in absolute units). Measured (**h**) and simulated (**i**) total gain (in dB).

We investigated the possibility of laser amplification of an ultrabroadband signal in a Tm^3+^-doped tellurite fiber simultaneously at two laser transitions ^3^F_4_ → ^3^H_6_ and ^3^H_4_ → ^3^H_5_ (see Fig. 1(a)). The scheme of the experimental setup is shown in Fig. 4(a). As a seed signal for the Tm^3+^-doped amplifier, we used supercontinuum generated in a nonlinear highly GeO_2_-doped silica fiber pumped by an all-fiber femtosecond Er laser system. The Er system consisted of a femtosecond passively mode-locked Er:fiber oscillator, a polarization controller, and a single-mode diode-pumped Er-doped amplifier. The amplified 2-nJ 70-fs pulses were coupled into a 2.5 m long fiber with 97 mol.% GeO_2_ content in the core. Generation of supercontinuum in a similar scheme has been already realized earlier^32,33^. The supercontinuum spectrum measured with a spectral filter (germanium plate transparent for the wavelengths above 1.7 μm) is shown in Fig. 4(b) by the grey curve. In numerical simulation, we approximated the measured spectrum by the red curve, which is the sum of four Gaussians (see Fig. 4(b)). The GeO_2_-doped fiber was butt-coupled to the Tm^3+^-doped tellurite fiber with length *L* = 2.2 m. The supercontinuum power launched into the core was estimated to be ~1.5 mW. For pumping the Tm^3+^-doped tellurite fiber, we used a multimode diode laser at 792 nm, having a fiber output with a core diameter of 105 μm and a numerical aperture of 0.22. The back-propagated pump was launched into a cladding with a diameter of 100 μm using a beam splitter (BS) and two aspheric lenses. Dielectric-coated glass BS was optimized for maximum reflection of the pump light at 792 nm and reflected about 8% at 1.9 μm and 2.3 μm due to Fresnel reflection. The backward pump configuration is, firstly, easier to implement, and, secondly, as will be shown later by numerical simulation, provides a higher efficiency and tolerance to the active fiber length in comparison with the forward pump configuration. The fiber ends were cleaved using an automatic ultrasonic cleaver (Fujikura CT-101) and inspected by an optical microscope before installation into the experimental setup. The pumped fiber end was cleaved at an angle of about 6 deg to avoid back-reflection of the pump light into the laser diode. The measured power of the amplified supercontinuum propagating in the core as a function of the launched pump power is shown in Fig. 4(c) by black circles. These measurements are in a perfect agreement with the results of numerical simulation. Ultrabroadband Tm^3+^-doped tellurite fiber amplifier was simulated by means of a well-tested home-made computer code. In brief, the code is based on a self-consistent solution of the following equations: rate equations taking into account the rates of the stimulated transitions depending on pump and signal powers as well as a cross-relaxation process in the simplified energy levels scheme shown in Fig. 1(a); equations describing CW pump evolution; and equations describing spectral evolution of signal waves: s1 amplified at ^3^F_4_ → ^3^H_6_ transition (near 1.9 μm) and s2 amplified at ^3^H_4_ → ^3^H_5_ transition (near 2.3 μm). The numerical model is described in the section Methods in detail. The used cross-sections shown in Fig. 1(b) were obtained as follows. We took the luminescence spectra measured experimentally for the Tm-doped tellurite glass with a similar chemical composition from the papers^20,34^ and calculated the emission cross-sections σ_21_ and σ_43_ using the Füchtbauer-Ladenburg equation^35^, then the absorption cross-section σ_12_ and σ_34_ were found via the McCumber theory^36^.

The measured output spectra at different pump powers are shown in Fig. 4(d). We used a diaphragm to cut off the light propagating through the fiber cladding. Then we integrated the spectra over the wavelength (see Fig. 4(c), squares) and obtained a very good agreement with the experimental and numerical dependences of the output supercontinuum power on the pump power. Here and further, the numerically calculated output signal powers are corrected taking into account ~10% Fresnel reflection at the output fiber end. The simulated spectra also perfectly agree with the experimentally measured spectra (see Fig. 4(d,e)). Next, we calculated the On/Off gain for the ^3^H_4_ → ^3^H_5_ transition, dividing the output spectra with nonzero pump by the spectrum without pump. The experimental and simulated curves are shown in Fig. 4(f,g), respectively. Note that, when the signal propagates at wavelengths > 2.2 μm without pump, it is attenuated only because of the background loss. The total gain differs from the On/Off gain by *αL* for the ^3^H_4_ → ^3^H_5_ transition. As one can see from Fig. 4(f), the maximum measured On/Off gain is 10·log_10_(10) = 10 dB with a pump power of 2.6 W. So, the maximum total gain is estimated by taking into account background loss as 10 dB − 1.3 dB/m·2.2 m ≈ 7 dB. We calibrated the input experimental spectrum shown in Fig. 4(b) to the output spectrum of the supercontinuum without the pump shown in Fig. 4(d) by a black line also using data on background loss at wavelengths λ > 2.2 μm. By dividing the measured spectra at different pump powers by the calibrated input spectrum, we obtained the total gain shown in Fig. 4(h). The total gain agrees very well with the numerical results shown in Fig. 4(i). In Fig. 4(h) it is seen that to obtain a positive gain at 2.3 μm, the pump power should exceed 0.64 W. The maximum gain at 2.3 μm was 7 dB for a pump power of 2.6 W. The maximum gain at the ^3^F_4_ → ^3^H_6_ transition was 30 dB at 1.9 μm for a pump power of 2.2 W. The spectrum and gain near 1.9 μm was not measured for a pump power of 2.6 W due to the insufficient dynamic range of our spectrometer. One can see in Fig. 4(h,i) that the wavelength of the gain maximum at the ^3^F_4_ → ^3^H_6_ transition depends on the pump power, while the position of the gain maximum at the ^3^H_4_ → ^3^H_5_ transition is practically independent. This has a simple explanation. In the simulation, it was found that the population of level 3 is small because of its short lifetime, so the maximum gain is observed at the maximum of the emission cross-section. For the ^3^F_4_ → ^3^H_6_ transition, populations of both levels 1 and 2 affect the gain. The higher the pump power, the greater the population of level 2 and the smaller the population of level 1 are. Therefore, for relatively large powers, the gain maximum is closer to the maximum of the emission cross-section, and at low powers, the maximum gain is shifted to the long-wavelength region.

**Figure 5:**
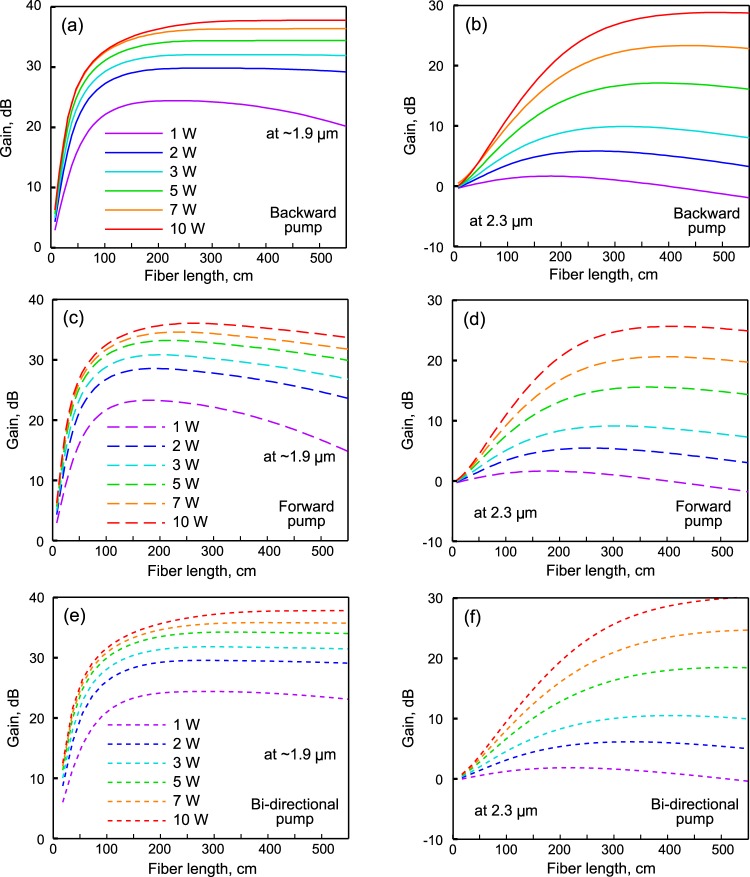
Simulated maximum total gain as a function of fiber length for backward pumped Tm-doped amplifier at ^3^F_4_ → ^3^H_6_ transition (**a**) and at ^3^H_4_ → ^3^H_5_ transition (**b**). Simulated maximum total gain as a function of fiber length for forward pumped Tm-doped amplifier at ^3^F_4_ → ^3^H_6_ transition (**c**) and at ^3^H_4_ → ^3^H_5_ transition (**d**). Simulated maximum total gain as a function of fiber length for bi-directional pumped Tm-doped amplifier at ^3^F_4_ → ^3^H_6_ transition (**e**) and at ^3^H_4_ → ^3^H_5_ transition (**f**).

The numerical results are in a very good agreement with experimental ones, so the developed theoretical model can be used to optimize amplifiers based on the developed Tm^3+^-doped tellurite fiber. We simulated the forward, backward, and bi-directional (50% forward & 50% backward) pump configurations for amplifiers of different lengths. We assumed that the pump power can be increased up to 10 W (which can be realized with commercially available multimode diode lasers with a power of 16–30 W). Further power increasing can lead to fiber damage^22^, although the damage threshold of the produced sample is not known to us. Figure 5 shows the dependence of the maximum gain at the ^3^F_4_ → ^3^H_6_ and ^3^H_4_ → ^3^H_5_ transitions on the fiber length. For pump powers of 1–2 W, the optimal fiber length is 2–3 m for the backward pump configuration for dual-band amplification at both transitions. Based on these numerical results, we chose the amplifier length in the experiment. As the pump power increases, the optimal amplifier length also increases, since the pump is absorbed over a longer distance. For example, at a power of 5–10 W, the amplifier length should be >4 m. With a pump power of 5–10 W, to obtain optimal amplification at a wavelength of 2.3 μm, the fiber length should be 4–6 m. One can see in Fig. 5 that the backward pump provides a higher gain in comparison with the forward pump. In addition, the optimal fiber lengths for amplification at both ^3^F_4_ → ^3^H_6_ and ^3^H_4_ → ^3^H_5_ transitions are almost the same for the backward pump, while for the forward pump, optimal lengths are different. Thus, the backward pump configuration makes it possible to obtain a higher gain in comparison with the forward pump configuration simultaneously at both transitions. For the bi-directional configuration, the gain is higher than for uni-directional ones because pump power is absorbed better and its distribution along the fiber axis is more uniform. However, it is more difficult to implement bi-directional pump configuration in experiment.

### Experimental and theoretical study of two-color CW laser based on Tm^3+^-doped TZLN fiber

**Figure 6:**
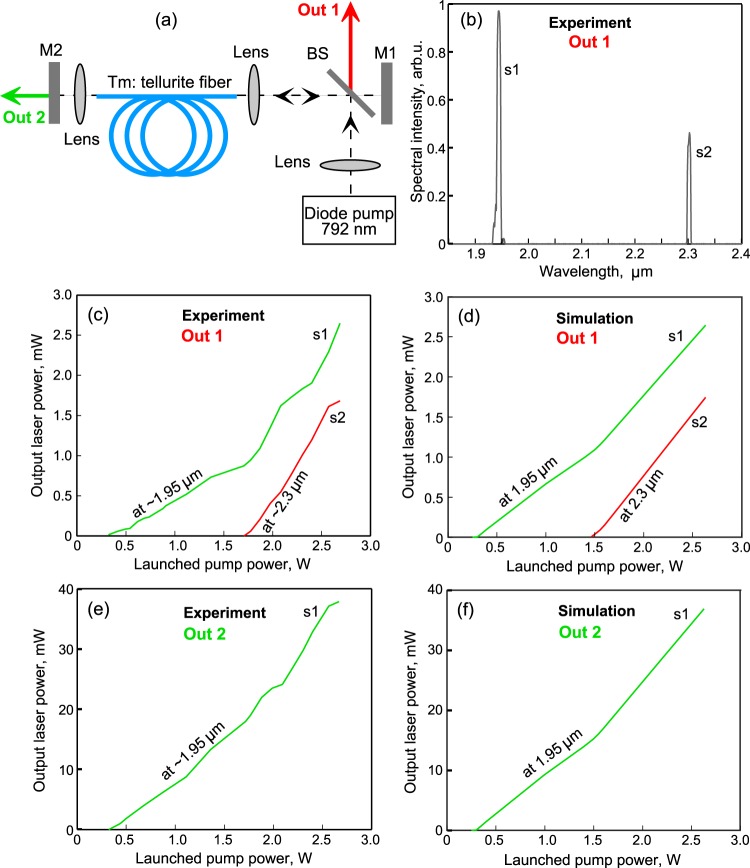
(**a**) Experimental setup of two-color CW laser. (**b**) Measured spectrum of laser signal for launched pump power of 2.2 W. Measured (**c**) and simulated (**d**) laser powers as a function of launched pump power at output 1. Measured (**e**) and simulated (**f**) laser power as a function of launched pump power at output 2.

Next, we investigated experimentally and theoretically a possibility of creating a two-color CW laser. We used the same Tm^3+^-doped fiber and pump as for the amplifier. The scheme of the experiment is shown in Fig. 6(a). Two mirrors were used to form a laser resonator: a highly reflective gold-coated mirror M1 (reflectivity 98%) and a dielectric mirror M2 highly reflective at 2.3 μm and partially transparent in the 1.8–2 μm range (reflectivity about 98%@2.3 μm and 30%@1.9 μm). Two-color radiation was outcoupled through BS (Out 1). The radiation at a wavelength of about 1.9 μm was also outcoupled through mirror M2 (Out 2). The two-color laser spectrum measured experimentally at Out 1 with a pump power of 2.2 W is shown in Fig. 6(b). The experimental output power *P*_*s1*_ in the signal wave s1 at the ^3^F_4_ → ^3^H_6_ transition and the output power *P*_*s2*_ in the signal wave s2 at the ^3^H_4_ → ^3^H_5_ transition as a function of pump power are plotted in Fig. 6(c). Using a monochromator, we divided s1 and s2 and measured their relative powers (*P*_*s1*_^*rel*^ and *P*_*s2*_^*rel*^, respectively). Knowing the absolute total power *P*_*Σ*_ directly at Out 1 we calculated absolute values *P*_*s1*_ = *P*_*Σ*_·*P*_*s1*_^*rel*^ /(*P*_*s1*_^*rel*^ + *P*_*s2*_^*rel*^) and *P*_*s2*_ = *P*_*Σ*_·*P*_*s2*_^*rel*^ /(*P*_*s1*_^*rel*^ + *P*_*s2*_^*rel*^). The experimental laser thresholds were 0.3 W and 1.7 W at the ^3^F_4_ → ^3^H_6_ and ^3^H_4_ → ^3^H_5_ transitions, respectively. We also performed numerical simulation using actual fiber parameters. The laser model is described in the section Methods in detail. Although, the transmission and reflection coefficients of the laser mirrors and the beam-splitter were known, as well as the transmission of the lenses (lens transmittance about 95%@1.9 μm and 85%@2.3 μm), additional losses caused by imperfect coupling of the light reflected from the mirror back into the fiber could not be easily measured. We did not know exactly the reflection and transmission coefficients in our system, and we took the following values for the estimation: *R*_*s1*_(*L*) = *R*_*s2*_(*L*) = 0.25, *R*_*s1*_(0) = 0.2, *R*_*p*_(0) = *R*_*s2*_(0) = 0.4 where *R*_*s1*_(*L*) and *R*_*s2*_(*L*) are the reflection coefficients of waves s1 and s2 at the output Out 1, *R*_*s1*_(0) and *R*_*s2*_(0) are the reflection coefficients of waves s1 and s2 at the output Out 2, *R*_*p*_(0) is the reflection coefficient of the pump at the output of Out 2 (see Fig. 6(a)). We made use of the transmission coefficients of waves s1 and s2 at the output Out 1 *T*_*s1*_(*L*) and *T*_*s2*_(*L*): *T*_*s1*_(*L*) = *T*_*s2*_(*L*) = 0.03. The transmission coefficients of waves s1 and s2 at the output Out 2 *T*_*s1*_(0) and *T*_*s2*_(0) were *T*_*s1*_(0) = 0.35, *T*_*s2*_(*L*) = 0. Note that *R*_*s1,s2*_(0, *L*) + *T*_*s1,s2*_(0, *L*) ≠ 1 because of additional losses in the system. The large losses were due to the use of non-optimal optical elements and imperfect free space-to-fiber light coupling. Thus, at the output of the fiber *z* = *L* (Out 1), there were at least the following losses: ~10% for Fresnel reflection from the fiber end, ~8% on the lens in the 1.9–2.3 μm range and ~8% on BS (Fresnel reflections of ~4% from each surface), ~2% on the gold mirror, then again losses on the BS, lens and end of the fiber (see Fig. 6(a)). Additional losses also occured when the reflected radiation from the resonator mirrors returned to the active fiber. The results of laser simulation with such parameters are shown in Fig. 6(d). The experimental and theoretical curves are in a good agreement. Note that after reaching the laser threshold for s2, a change in the behavior of the curve for s1 is observed. The slope efficiency for s1 increases due to the increase in the population of level 2 via the decrease in the population of level 4. We also measured the dependence of the power s1 at Out 2 on the pump power (see Fig. 6(e)) and simulated this case (see Fig. 6(f)). The experimental and theoretical curves are in a good agreement.

To demonstrate the advantages (lower laser threshold and higher slope efficiency at 2.3 μm) of the two-color scheme in comparison with the single-color one, we considered theoretically lasing of the wave s2 only. Lasing of the wave s1 was suppressed by introducing additional selective losses in the 1.8–2 μm spectral range. Other parameters of the modeled scheme did not change. We found that the laser threshold at 2.3 μm increased by ~30% (in comparison with the laser threshold shown in Fig. 6(d)) and the slope efficiency decreased by ~4 times. The explanation is that in the absence of s1 generation, the population of level 1 decreases (due to the accumulation of electrons at level 2), which reduces the population of level 4 (approximately in proportion to the population of level 1).

**Figure 7:**
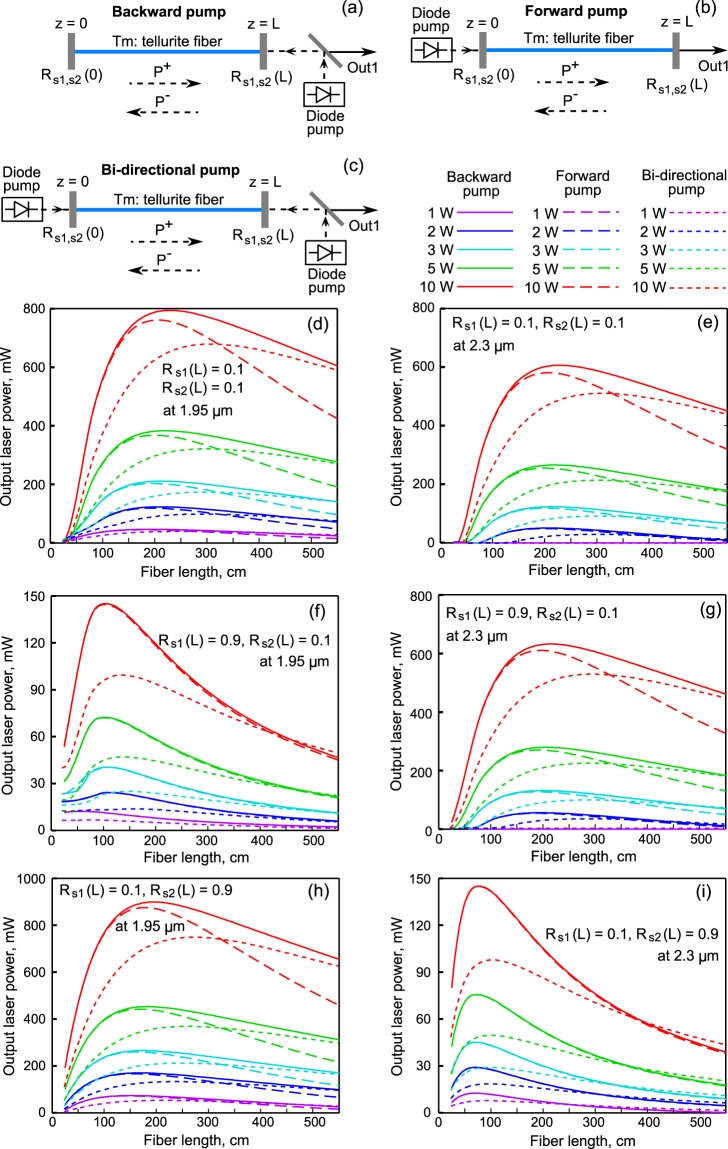
Modeled scheme of backward (**a**), forward (**b**), and bi-directional (**c**) pumped two-color laser. Simulated signal powers for different output reflection coefficients R_s1,s2_(L) for wave s1 at 1.95 μm (**d**,**f**,**h**) and for wave s2 at 2.3 μm (**e**,**g**,**i**).

Further, we investigated theoretically the possibility of increasing the efficiency of laser generation by optimizing the optical scheme, which can be done with the current technology level. We tried to find a global maximum of output power at 2.3 μm in the produced active fiber (for fixed values of Tm^3+^ ion concentration, optical loss, core and cladding diameters). We considered the forward, backward, and bi-directional pump configurations (see Fig. 7(a–c) respectively). For uni-directional pump configurations, we assumed full pump wave reflection from the opposite fiber end, but for the bi-directional pump, we assumed that pump waves were not reflected from both fiber ends. We assumed that at one end of the fiber the reflection coefficient for signal waves can be close to 1 (*R*_*s1,s2*_(0) = 1), which in principle can be realized, for example, by using a Bragg grating or by depositing a dielectric mirror at the fiber end^26^. The reflection coefficients *R*_*s1*_(*L*) and *R*_*s2*_(*L*) at Out 1 for waves s1 and s2 were independently varied in the 0.01–0.99 range and losses at mirrors were neglected (*R*_*s1,s2*_(*L*) + *T*_*s1,s2*_(*L*) = 1). We made sure, that laser generation at the ^3^H_4_ → ^3^F_4_ radiative transition near 1.4 μm was not observed in our study (because the population of level ^3^F_4_ was noticeably higher than the population of level ^3^H_4_). We simulated two-color lasers for different pump powers and Tm fiber lengths. Thus, we varied all the main parameters which affect the efficiency of laser generation.

One can see in Fig. 7 the calculated output laser powers as functions of the active fiber length for the three following combinations of reflection coefficients: 1) *R*_*s1*_(*L*) = 0.1 and *R*_*s2*_(*L*) = 0.1 (Fig. 7(d) for s1 and Fig. 7(e) for s2); 2) *R*_*s1*_(*L*) = 0.9 and *R*_*s2*_(*L*) = 0.1 (Fig. 7(f) for s1 and Fig. 7(g) for s2); as well as 3) *R*_*s1*_(*L*) = 0.1 and *R*_*s2*_(*L*) = 0.9 (Fig. 7(h) for s1 and Fig. 7(i) for s2). At low reflection coefficients, it is preferable to use the backward pump configuration, whereas for large reflection coefficients the difference between forward and backward configurations is very small, since the power distributions of the corresponding signal wave along *z* inside the fiber are almost the same at almost identical boundary conditions at *z* = 0 and *z* = *L*. Note an interesting fact tested for a wide range of parameters: for two-color generation, the output power of wave s2 at 2.3 μm for a fixed value of *R*_*s2*_(*L*) is practically independent of *R*_*s1*_(*L*). By way of illustration, Fig. 7(e,g) demonstrate that despite the fact that in one case the reflection coefficient is small at 1.95 μm (*R*_*s1*_(*L*) = 0.1) and in the other case large (*R*_*s1*_(*L*) = 0.9), the curves corresponding to the same pump powers and pump configurations for s2 behave almost identically; the difference in the output powers of s2 near the maxima is less than 10% (but a slightly higher efficiency is achieved at large *R*_*s1*_(*L*)). If we fix *R*_*s1*_(*L*) and change *R*_*s2*_(*L*) (compare Fig. 7(d,h)), we can see a similar trend: the behavior of the curves for s1 depends little on *R*_*s2*_(*L*) (but a slightly higher efficiency for s1 is achieved for large *R*_*s2*_(*L*)). Uni-directional pump configurations are preferable due to higher absorbed pump powers in comparison with bi-directional configuration (we assumed that mirrors from which pump was launched into the fiber were transparent at 792 nm and also that opposite mirrors had 100% reflectivity at 792 nm for forward and backward configurations).

**Figure 8:**
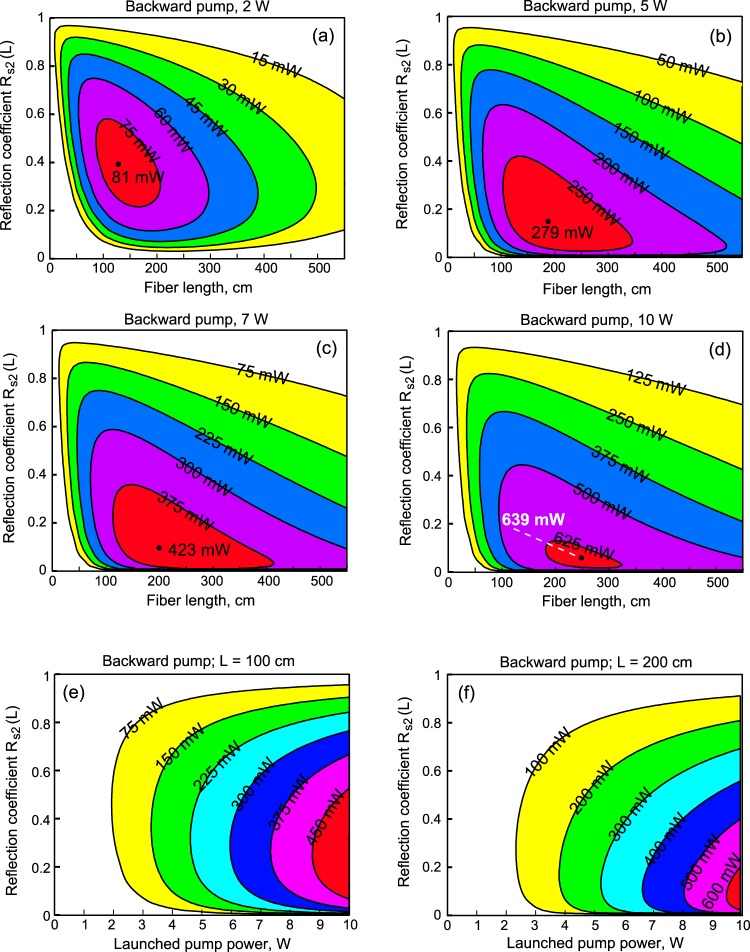
Simulated power of laser signal at 2.3 μm as a function of output reflection coefficient R_s2_(L) and fiber length for R_s1_(L) = 0.99 for launched pump powers of 2, 5, 7, and 10 W (**a**–**d**), respectively. Simulated power of laser signal at 2.3 μm as a function of output reflection coefficient R_s2_(L) and launched pump power for R_s1_(L) = 0.99 and L = 100 cm (**e**), L = 200 cm (**f**).

Next, we fixed *R*_*s1*_(*L*) = 0.99 and plotted the output power of wave s2 at 2.3 μm as a function of two variables: the active fiber length and the output reflection coefficient *R*_*s2*_(*L*) for the backward pump configuration. The level lines of *P*_*s2*_(*L*, *R*_*s2*_(*L*)) for the powers of 2, 5, 7, and 10 W are shown in Fig. 8(a–d), respectively. The higher the pump power, the lower the optimal reflection coefficient and the longer the optimal length are. Thus, for a pump power of 10 W, the maximum output power of 639 mW at 2.3 μm is obtained for *R*_*s2*_(*L*) = 0.05 and *L* = 250 cm; for pump powers of 7, 5, 2 W, the maximum output powers of 423, 279, 81 mW are obtained for *R*_*s2*_(*L*) = 0.1 and *L* = 200 cm, *R*_*s2*_(*L*) = 0.15 and *L* = 190 cm, *R*_*s2*_(*L*) = 0.4 and *L* = 125 cm, respectively.

Then, we also fixed *R*_*s1*_(*L*) = 0.99 and plotted the output power of wave s2 at 2.3 μm as a function of two other variables: the pump power and the output reflection coefficient *R*_*s2*_(*L*) for the case of the backward pump configuration. The level lines of *P*_*s2*_(*P*_*p*_, *R*_*s2*_(*L*)) for the active fiber length of 100 cm and 200 cm are shown in Fig. 8(e,f), respectively. For length *L* = 200 cm, the maximum slope efficiency of ~7.5% is obtained for *R*_*s2*_(*L*) = 0.05. For these parameters, the laser threshold is ~1.5 W, and for *P*_*p*_ = 10 W, the maximum output power is 629 mW. Saturation begins to influence *P*_*s2*_ when P_p_ ≥ 9 W. For the length *L* = 100 cm, the maximum slope efficiency of ~6% is obtained for *R*_*s2*_(*L*) = 0.4. In this case, for *P*_*p*_ = 10 W, the maximum output power is 512 mW. Starting from *P*_*p*_ ≈ 7 W, saturation begins to be noticeable.

For comparison, we also considered the case of the single-color generation of wave s2 by introducing large selective losses for wave s1 in the 1.8–2 μm range for the backward pump configuration. We found that the maximum output power at 2.3 μm is significantly smaller than the output power at 2.3 μm in the case of two-color lasing. For the single-color generation, the optimal active fiber is longer. For example, for *R*_*s2*_(*L*) = 0.1, the maximum output powers are: 26 mW at *L* = 330 cm, 52 mW at *L* = 430 cm, 69 mW at *L* = 470 cm and 85 mW at *L* = 530 cm for pump powers 3, 5, 7, and 10 W, respectively. So, these powers are indeed significantly less than the maximum powers obtained for two-color generation (see Fig. 7(d,f)). Thus, the two-color generation provides an effective depopulation of laser level ^3^F_4_ with a long lifetime, which leads to decreasing the laser threshold and a considerable increase in the efficiency of wave s2 at 2.3 μm. Besides, the dual-band scheme also reduces the parasitic thermooptical effects^37^ because level ^3^F_4_ is depopulated radiatively.

## Discussion and Conclusions

We developed and synthesized TeO_2_-ZnO-La_2_O_3_-Na_2_O glass stable against crystallization by a special technique, providing a low concentration of hydroxyl groups (~10^17^ cm^−3^). We produced a high-quality, low background loss (~1 dB/m in the 2–2.5 μm range) fiber with an 8-μm core doped with Tm^3+^ ions (with a concentration of 5·10^19^ cm^−3^). We demonstrated experimentally, for the first time to the best of our knowledge, ultrabroadband amplification simultaneously at the ^3^F_4_ → ^3^H_6_ and ^3^H_4_ → ^3^H_5_ laser transitions in Tm^3+^-doped tellurite fiber. The maximum gain of 30 dB and 7 dB was measured at 1.9 μm and 2.3 μm, respectively, with the backward pump by a multimode laser diode at 792 nm. As a seed for an amplifier, we used supercontinuum in the 1.7–2.8 μm range from a highly GeO_2_ doped silica fiber pumped by an Er fiber femtosecond laser system. The seeding signal power launched in the core was ~1.5 mW. The maximum average power of the amplified signal was 80 mW. Two-color CW lasing at 1.95 and 2.3 μm was also obtained for the first time in Tm^3+^-doped tellurite fiber with maximum powers of 2.7 mW and 1.7 mW, respectively, at one output and a maximum power of ~40 mW at 1.95 μm at the other output. The maximum pump power launched in 100-μm cladding was ~2.6 W. The low efficiency was explained by non-optimal optical elements available to us. However, the achieved laser powers are sufficient for some applications, for example, gas detection (CH_4_ at 2.3 μm^2^ or CO_2_ at 1.95 μm^38^).

We also presented detailed experimental and theoretical studies, which are in a very good agreement, of laser amplification and generation in the manufactured fiber with carefully measured and calculated parameters. A quantitatively verified numerical model was used to predict power scalability at 2.3 μm in dual-band schemes with optimized parameters under increased pump power. It was shown numerically that the maximum gain at 2.3 μm can reach 30 dB in an amplifier with the bi-directional 10-W pump (which is preferable to uni-directional ones). Maximum simulated power at 2.3 μm for two-color laser exceeded 600 mW for backward 10-W pump. We also showed theoretically that in the case of generating only the wave at the ^3^H_4_ → ^3^H_5_ transition (by adding very large selective losses for the 1.8–2 μm spectral range), the maximum output power at 2.3 μm is significantly (an order of magnitude) smaller than the output power at 2.3 μm in the case of dual-band operation. This has a simple explanation. Lasing at 2.3 μm from the ^3^H_4_ → ^3^H_5_ transition suffers a bottleneck problem from the ~10 times larger lifetime of electrons at the ^3^F_4_ level as compared to the ^3^H_4_ level, slowing down electrons’ return to the ground level. Co-lasing at 1.9 μm helps to depopulate the ^3^F_4_ level. Besides, dual-band lasing also reduces the parasitic thermo-optical effects in comparison with lasing only at 2.3 μm because the laser level ^3^F_4_ is depopulated radiatively.

So, the present study demonstrates that a high-quality Tm^3+^-doped tellurite fiber is a promising novel gain element for lasers and amplifiers in the atmospheric window near 2.3 μm, which may be a good alternative to other active media in this spectral range, in particular, to Tm^3+^-doped fluoride fibers.

## Methods

### Numerical model for Tm^3+^-doped tellurite fiber amplifier and laser

To simulate a Tm-doped ultrabroadband fiber amplifier and a two-color CW laser we used the following equations. The rate equations for the population densities *n*_*1*_, *n*_*2*_, *n*_3_, *n*_4_ (normalized to the concentration of Tm^3+^ ions in the core *N*_*Tm*_ = 5·10^19^ cm^−3^) are given by^29^:

1 $$\frac{\partial {n}_{4}}{\partial t}={W}_{14}{n}_{1}+{W}_{34}{n}_{3}-({W}_{41}+{W}_{43}+\frac{1}{{\tau }_{4}}){n}_{4}-{K}_{CR}{n}_{4}{n}_{1}=0,$$

2 $$\frac{\partial {n}_{3}}{\partial t}=-({W}_{34}+\frac{1}{{\tau }_{3}}){n}_{3}+({W}_{43}+\frac{1}{{\tau }_{4}^{NR}}+\frac{{\beta }_{43}}{{\tau }_{4}^{R}}){n}_{4}=0,$$

3 $$\frac{\partial {n}_{2}}{\partial t}={W}_{12}{n}_{1}-({W}_{21}+\frac{1}{{\tau }_{2}}){n}_{2}+\frac{{n}_{3}}{{\tau }_{3}}+\frac{{\beta }_{42}}{{\tau }_{4}^{R}}{n}_{4}+2{K}_{CR}{n}_{4}{n}_{1}=0,$$

4 $${n}_{1}+{n}_{2}+{n}_{3}+{n}_{4}=1,$$

where *τ*_*4*_, *τ*_*4*_^*R*^ and *τ*_*4*_^*NR*^ are the total, radiative and non-radiative lifetimes of level 4, respectively (*τ*_*4*_ = 0.3 ms^20,34^, *τ*_*4*_^*R*^ = 0.4 ms^29^, *τ*_*4*_^*NR*^ = (1/*τ*_*4*_ − 1/*τ*_*4*_^*R*^)^−1^ = 1.2 ms); *τ*_*3*_ is the total (non-radiative) lifetime of level 3 (*τ*_*3*_ = 0.13 μs^20,34^); *τ*_*2*_ is the total lifetime of level 2 (*τ*_*2*_ = 3 ms^20,24,29^); *β*_*4y*_ is the branching ratio from level 4 to level *y* = 1, 2, 3 (*β*_*41*_ = 0.9, *β*_*42*_ = 0.07, *β*_*43*_ = 0.03^29^); *K*_*CR*_ is the coefficient of cross-relaxation (*K*_*CR*_ = 1000 s^–1^ ^29^), *W*_*xy*_ are the stimulated rates. Stimulated rates by hydroxyl groups were neglected due to their extremely low concentration in the produced sample. For an amplifier, absorption and emission stimulated rates of the forward pump with power *P*_*p*_^+^ and/or backward pump with power *P*_*p*_^*−*^ at wavelength *λ*_*p*_ = 0.792 μm are

5 $${W}_{14,41}=\frac{{{\rm{\Gamma }}}_{p}{\lambda }_{p}{\sigma }_{14,41}({\lambda }_{p})}{hc{A}_{core}}({P}_{p}^{+}+{P}_{p}^{-})$$

where *h* is Planck’s constant; *c* is the speed of light; *A*_*core*_ ≈ 50 μm^2^ is the doped core area, *σ*_*14*_ and *σ*_*41*_ are the absorption and emission cross sections at *λ*_*p*_ (*σ*_*14*_ = *σ*_*41*_ = 1·10^−20^ cm^2^); *Γ*_*p*_ is the overlap integral of the pump intensity distribution with the core estimated as the ratio of the core area to the cladding area (*Γ*_*p*_ = 0.064). We consider a quasi-steady-state amplification of ultrabroadband pulses with repetition rate *v* = 50 MHz, so equations (1)–(3) are averaged over time 1/*v*, assuming that the populations slightly vary for this period. As a result, the time derivatives on the left-hand side of equations (1)–(3) become equal to zero, and the expressions for the rates of the signal-stimulated transitions at the central wavelengths *λ*_*s1*_ and *λ*_*s2*_ are written as^39^

6 $${W}_{12,21}=\frac{{{\rm{\Gamma }}}_{s1}{\lambda }_{s1}}{hc{A}_{core}}{v}{\int }_{1.5\mu m}^{2.15\mu m}{\sigma }_{12,21}(\lambda )|{A}^{+}(\lambda ){|}^{2}d\lambda $$

7 $${W}_{34,43}=\frac{{{\rm{\Gamma }}}_{s2}{\lambda }_{s2}}{hc{A}_{core}}{v}{\int }_{2.15\mu m}^{2.55\mu m}{\sigma }_{34,43}(\lambda )|{A}^{+}(\lambda ){|}^{2}d\lambda $$

where the emission and absorption cross-sections *σ*_*21,43*_ and *σ*_*12,34*_ are taken from Fig. 1(b); *Γ*_*s1*_ and *Γ*_*s2*_ are the overlap integrals of the signal intensity distribution with core estimated on the basis of LP_01_ mode calculation (*Γ*_*s1*_ = *Γ*_*s2*_ = 0.8), *A*^+^(*λ*) is the spectral amplitude of the forward propagating signal. The equations describing the evolution of the pump and signal waves propagating along the z-axis are

8 $$\mp \frac{d{P}_{p}^{\pm }}{dz}={{\rm{\Gamma }}}_{p}{N}_{Tm}[{\sigma }_{14}({\lambda }_{p}){n}_{1}-{\sigma }_{41}({\lambda }_{p}){n}_{4}]{P}_{p}^{\pm }+\alpha ({\lambda }_{p}){P}_{p}^{\pm }$$

9 $$\frac{\partial {A}^{+}(z,\lambda )}{\partial z}=\frac{-\alpha (\lambda )}{2}{A}^{+}(z,\lambda )+\frac{g(\lambda )}{2}{A}^{+}(z,\lambda ),$$

*α*(*λ*) is background loss; *g*(*λ*) describes stimulated amplification and absorption at both ^3^F_4_ → ^3^H_6_ and ^3^H_4_ → ^3^H_5_ transitions:

10 $$g(\lambda )={{\rm{\Gamma }}}_{s1}{N}_{Tm}[{\sigma }_{21}(\lambda ){n}_{2}-{\sigma }_{12}(\lambda ){n}_{1}]+{{\rm{\Gamma }}}_{s2}{N}_{Tm}[{\sigma }_{43}(\lambda ){n}_{4}-{\sigma }_{34}(\lambda ){n}_{3}]$$

The average signal power is

11 $$\langle {{P}_{s}}^{+}\rangle ={v}\int |{A}^{+}(\lambda ){|}^{2}d\lambda $$

Here we neglected the nonlinear effects due to low peak powers. But they can be easily incorporated in the developed model^39,40^. In the case of highly chirped pulses with high peak power, specially developed numerical algorithms can be also used for effective modeling^41,42^.

For a CW laser, signal rates of the stimulated transitions are written in the form^43^:

12 $${W}_{12,21}=\frac{{{\rm{\Gamma }}}_{s1}{\lambda }_{s1}{\sigma }_{12,21}({\lambda }_{s1})}{hc{A}_{core}}({P}_{s1}^{+}+{P}_{s1}^{-})$$

13 $${W}_{34,43}=\frac{{{\rm{\Gamma }}}_{s2}{\lambda }_{s2}{\sigma }_{34,43}({\lambda }_{s2})}{hc{A}_{core}}({P}_{s2}^{+}+{P}_{s2}^{-})$$

where *P*_*s1,s2*_^+^ and *P*_*s1,s2*_^*−*^ are the powers of forward and backward propagating waves, respectively (see Fig. 7(a,b)). Pump rates of the stimulated transitions are described by Eq. (5). For the pump powers evolution we use Eq. (8). The power evolution in signal waves is given by^43^:

14 $$\pm \frac{d{P}_{s1}^{\pm }}{dz}={{\rm{\Gamma }}}_{s1}{N}_{Tm}[{\sigma }_{21}({\lambda }_{s1}){n}_{2}-{\sigma }_{12}({\lambda }_{s1}){n}_{1}]{P}_{s1}^{\pm }-\alpha ({\lambda }_{s1}){P}_{s1}^{\pm }$$

15 $$\pm \frac{d{P}_{s2}^{\pm }}{dz}={{\rm{\Gamma }}}_{s2}{N}_{Tm}[{\sigma }_{43}({\lambda }_{s2}){n}_{4}-{\sigma }_{34}({\lambda }_{s2}){n}_{3}]{P}_{s2}^{\pm }-\alpha ({\lambda }_{s2}){P}_{s2}^{\pm }$$

The boundary conditions are the following:

16 $${P}_{s1}^{+}(0)={R}_{s1}(0){P}_{s1}^{-}(0),\,{{P}_{s1}}^{-}(L)={R}_{s1}(L){P}_{s1}^{+}(L)$$

17 $${P}_{s2}^{+}(0)={R}_{s2}(0){P}_{s2}^{-}(0),\,{{P}_{s2}}^{-}(L)={R}_{s2}(L){P}_{s2}^{+}(L)$$

The output laser powers of waves s1 and s2 at out1 (see Fig. 6(a) and Fig. 7(a,b)) and laser power of waves s1 at out2 (see Fig. 6(a)) are

18 $${P}_{s1,s2}^{out1}={T}_{s1,s2}(L){P}_{s1,s2}^{+}(L),\,{P}_{s1}^{out2}(0)={T}_{s1}(0){P}_{s1}^{-}$$

Here, *T*_*s1,s2*_(*L*) and *T*_*s1*_(0) are the transmission coefficients at *z* = *L* and *z* = 0, respectively.

To simulate pump and signal evolution we used the fourth order Runge-Kutta method. For backward pumped amplifiers to meet the boundary conditions *A*_*s1,s2*_^+^(0) and *P*_*p*_^*−*^(*L*) we applied the shooting method. For the lasers to meet the boundary conditions (16)–(18), a fixed point iteration method was employed^43^.
